# Dealing with treatment and transfer requests: how PGD-professionals discuss ethical challenges arising in everyday practice

**DOI:** 10.1007/s11019-017-9811-0

**Published:** 2017-10-28

**Authors:** Melisa Soto-Lafontaine, Wybo Dondorp, Veerle Provoost, Guido de Wert

**Affiliations:** 1 Department of Health Ethics & Society, CAPHRI Care and Public Health Research School, GROW School for Oncology and Developmental Biology, Maastricht University, PO Box 616, 6200 MD Maastricht, The Netherlands; 2 Bioethics Institute Ghent, Ghent University, Blandijnberg 2, 9000 Ghent, Belgium

**Keywords:** Preimplantation genetic diagnosis (PGD), Indications, Embryo selection, Ethics, Stakeholder views

## Abstract

How do professionals working in pre-implantation genetic diagnosis (PGD) reflect upon their decision making with regard to ethical challenges arising in everyday practice? Two focus group discussions were held with staff of reproductive genetic clinics: one in Utrecht (The Netherlands) with PGD-professionals from Dutch PGD-centres and one in Prague (Czech Republic) with PGD-professionals working in centres in different European countries. Both meetings consisted of two parts, exploring participants’ views regarding (1) treatment requests for conditions that may not fulfill traditional indications criteria for PGD, and (2) treatment and transfer requests involving welfare-of-the-child considerations. There was general support for the view that people who come for PGD will have their own good reasons to consider the condition they wish to avoid as serious. But whereas PGD-professionals in the international group tended to stress the applicants’ legal right to eventually have the treatment they want (whatever the views of the professional), participants in the Dutch group sketched a picture of shared decision-making, where professionals would go ahead with treatment in cases where they are able to understand the reasonableness of the request in the light of the couple’s reproductive history or family experience. In the international focus group there was little support for guidance stating that welfare-of-the child considerations should be taken into account. This was different in the Dutch focus group, where shared decision-making also had the role of reassuring professionals that applicants had adequately considered possible implications for the welfare of the child.

## Introduction

Since preimplantation genetic diagnosis (PGD) was introduced in the early 1990s, the use of this technology has become an established reproductive option in many countries. PGD was developed to help prospective parents at a known high risk of transmitting a serious monogenetic disorder, to have healthy children. Soon, PGD was also offered to prospective parents carrying a chromosomal abnormality (translocation) that may either lead to (repeated) pregnancy loss or to the birth of a severely handicapped child (Collins 2013; Geraedts and De Wert 2009).

PGD requires a step of assisted fertilisation in the lab in order to obtain embryos that can then be analysed in vitro. This makes PGD a demanding procedure for the couple, especially for the woman who, despite being fertile in most cases, has to undergo the hormonal treatment necessary for harvesting several mature oocytes. After fertilization through intracytoplasmic sperm injection (ICSI; a form of in vitro fertilization: IVF), the resulting embryos (of good enough quality) are subjected to a biopsy at the cleavage or (increasingly) at the blastocyst stage. The cells taken from the embryo are analysed for the presence or absence of the relevant mutation or chromosome abnormality. In most cases, depending on the type of abnormality, those cells can safely be regarded as representative for the embryo as a whole. This then allows the final step of the selecting only embryos unaffected by the relevant mutation or abnormality for transfer to the womb.

As compared to the alternative option of prenatal diagnosis, PGD has the advantage of preceding the establishment of pregnancy and thus to spare the couple difficult decision making about whether or not to continue the pregnancy if the foetus is diagnosed with the condition for which they are at risk. However, the fact that PGD involves selecting embryos for transfer is ethically sensitive and has put the technology in the centre of societal debate about assisted reproductive technologies (Knoppers et al. 2006). Whereas on the one hand the acceptance of PGD is growing (with more countries allowing the technology under conditions), on the other hand scientific developments in this field keep raising concerns about ‘where to draw the line’ (Hens et al. 2013; Vermeesch et al. 2016). Examples of possible applications that many find troubling are PGD for sex selection (except to avoid a sex-linked disorder) and PGD to select a child with a handicap (e.g., deaf parents wanting a deaf child) (Davis 2010).

However, ethical issues that arise in everyday PGD-practice (and can be said to be more important for that reason) have attracted less attention (De Wert et al. 2014b). In the monthly meetings of the PGD-committee in our academic hospital in Maastricht, The Netherlands, two questions keep returning: (1) how to deal with treatment requests relating to conditions for which the severity or the level of risk may not be serious enough to warrant PGD? (2) how to deal with treatment or transfer requests that raise concerns about the welfare of the child that may be born as a result? The qualitative study reported in this paper explores the views of PGD-professionals on these issues. The study is part of a larger project aiming to contribute to the evaluation and fine-tuning of current Dutch PGD policy and guidance. In view of this aim, two focus group meetings were held. In order to find out how, within that current framework, PGD professionals in The Netherlands decide about PGD treatment-and transfer requests, a first focus group was held with professionals from all four PGD-centres in The Netherlands. Second, in order to allow putting the findings in a wider perspective, the same issues were discussed in a focus group with professionals working in PGD-centres in different European countries.

## Background

### PGD indications

In countries where PGD is available, its lawful application is often limited to what in a strict sense of the term, can be called ‘the medical model’(Geraedts and De Wert 2009). Under this model, PGD is an accepted treatment for couples (or individuals) at risk of transmitting a genetic disorder or handicap to their offspring, or of losing the pregnancy due a chromosomal disorder. In several European countries, (e.g. Denmark^1^, France^2^, Germany^3^, The Netherlands^4^, Norway^5^, Sweden^6^, and the UK^7^), the legislator has further qualified the medical model by stipulating that PGD requires a ‘significant’ or ‘high’ risk of bearing a child with a ‘serious’ genetic disorder. The relevant Spanish legislation^8^ only contains the latter requirement, but adds that the disorder must be incurable, a condition also found in the French Public Health Code, but not in most other jurisdictions.

Reasons why it is thought that PGD should only be offered for serious conditions are not always spelled out (De Wert et al. 2014b). The reasons given include the fact that embryo testing and selection requires burdensome and costly ICSI treatment (often, in part, subsidised from public or collective funds), the moral sensitivity of embryo selection, concerns that the procedure (including embryo biopsy) may have subtle adverse long-term health effects and the fear that allowing PGD for less serious conditions would be a step on a slippery slope towards the dreaded ‘designer child’ (Berthiau 2013; Finck et al. 2006; Steinkamp et al. 2012).

However, it is not immediately obvious how the criteria ‘significant/high risk’ and/or ‘serious disorder’ are to be understood and applied in practice. Apart from Germany and Norway, where each PGD case must be approved by a multidisciplinary ethics committee this is to a large extent left to the field to determine. This is most obviously the case in countries where no further mechanisms are in place to provide or guide this interpretation. But also where there are such mechanisms, as e.g., in The Netherlands and the UK (see “Discussion”), decisions with regard to particular cases will still have to be made by individual professionals and centres. In countries without specific legislation on PGD-indications, or where this is limited to a ban on non-medical applications (e.g., Belgium^9^), it is entirely up to professionals to determine for conditions of what level of risk or seriousness PGD should be available. This only makes the question more pertinent as to what they regard as acceptable reasons for PGD and on the basis of what considerations.

### Welfare of the child

According to the European Society of Human Reproduction and Embryology (ESHRE), professionals working in assisted reproduction have a responsibility to take account of the welfare of the child whose very existence they causally and intentionally bring about. More specifically, this means that professionals should refrain from providing assisted reproduction if there is a high risk of a child with a seriously diminished quality of life (Pennings et al. 2007). Similar guidance is given by e.g. the UK Human Fertilisation & Embryology Authority (HFEA) in its Code of Practice^10^ and the Dutch Society of Obstetrics & Gynaecology (NVOG)^11^. In the literature, this issue is mainly discussed with an eye to societal concerns about the welfare of the child in non-standard relationships or single parent families (De Wert et al. 2014a), and also with regard to concerns about creating families at a high risk of child abuse or neglect (Pennings 1999, 2011; Peterson 2005; Thompson and McDougall 2015; Hunfeld et al. 2004). However specific welfare-of-the-child concerns may arise in PGD practice which have not until now been given much attention in the literature on the ethics of PGD, nor in professional guidance documents. These concerns are of two kinds. Firstly, PGD may be requested to avoid the transmission of an untreatable later onset disorder of which one of the intended parents is expected to suffer and die during the child’s youth or adolescence, as may be the case with PGD for Huntington’s disease (de Die-Smulders et al. 2013). Secondly, welfare-of-the-child concerns may lead to situations of conflict between professionals and intended parents in cases where PGD has not led to any suitable embryos unaffected by the disorder that the procedure was meant to avoid. In such cases, professionals are sometimes confronted with a request to go ahead anyway and transfer an affected embryo (Thornhill et al. 2005). Our study is the first to report on professional views about how to handle such situations.

## Materials and methods

Two focus group discussions were held with staff of reproductive genetic clinics: 1 in Utrecht (The Netherlands) with 12 PGD-professionals from all 4 Dutch centres offering PGD and 1 in Prague (Czech Republic) with 12 PGD-professionals working in centres in different European countries with different health care settings (Belgium, Czech Republic, Germany, Greece, Hungary, Italy).

For both focus groups purposeful sampling was used in order to learn from professionals with relevant expertise in PGD treatment and experience with a wide range of PGD requests. Potential participants for the Dutch focus group were approached through open call emails addressed to all (four) PGD-centres in The Netherlands. For the international focus group, all professionals who registered for an educational symposium organised by the Special Interest Group on Reproductive Genetics of the European Society of Embryology and Human Reproduction (ESHRE), were personally invited by email. The background of participants of both focus groups covered the spectrum of professional groups dealing with PGD, both in the lab (embryology, molecular and cyto-genetics) and in patient care (gynaecology, clinical genetics, counselling). Eleven participants were male and 13 female.

Both meetings lasted around two hours and started with a brief introduction. The Dutch focus group was moderated by GdW; the international focus group by Kristien Hens. Each discussion consisted of two parts comprising the two questions outlined above. In both focus groups, cases were used to initiate a dynamic and reflective conversation. The first part explored participants’ views regarding acceptable reasons for PGD. Participants were invited to discuss cases, or present additional cases, of PGD-requests that challenge a strict interpretation of the ‘medical model’. The second part explored participants’ views regarding cases involving welfare-of-the-child considerations.

The discussion sessions were audiotaped and transcribed verbatim. Informants were anonymized with codes indicating the country where the discussions were held, the corresponding number of the excerpt and the speaker‘s gender. The transcripts were scrutinized through an interactive process for generating codes for recurrent themes. Following Braun and Clarke’s Inductive Thematic Analysis model (Braun and Clarke 2006), our coding process aimed to identify and analyse patterns to report on the participants’ experience, meanings and practices surrounding the ethics of PGD. Data were independently coded by the first two authors (MSL and WD). To improve the validity and the trustworthiness of the analysis, an auditing process was conducted. After the initial analysis, an auditor (VP) challenged the analysis of the first two authors to promote its depth and to identify and adjust discrepancies as well as gaps (Hill et al. 1997). Quotes (translated from Dutch to English for the first focus group) were used to illustrate the themes. For readability, some of the quotes in this report were edited.

## Results

Whereas the topic lists for the focus groups were designed to collect data on specific criteria that professionals considered relevant for PGD decision-making, the analysis of the data showed that first and foremost professionals unsolicited and spontaneously referred to the wishes and experiences of their patients as primary decision-makers. Although specific criteria were discussed and examined by the participants during the focus group discussions, their attention shift from those criteria to the patient as decision-maker was a recurrent finding. The fact that this was brought up unsolicited underlines the importance of this finding. In relation to several specific criteria, the participants stated that the assessment of a criterion could not be disentangled from the views and situation of the requesting patients.

### Reasons for PGD-requests

#### Severity of the disorder

In both focus groups, there was much support for the view that the seriousness of the disorder for which PGD is requested cannot be determined in isolation from the psychosocial impact as experienced by the applicants. Repeatedly, it was said that the mere fact that people are willing to accept the burdens (and costs) of PGD treatment to avoid the transmission of a specific condition, indicates that for them that condition is serious, even if this seriousness would not seem obvious either in terms of the severity of the disorder per se, or in view of co-determinants such as a lower penetrance and/or variable expression (see below). To illustrate this for severity per se, a participant in the Dutch focus group came up with the following case.

Only last week we had a discussion concerning a patient suffering from congenital hair loss. You may say that is nothing, but for the patient this was unbelievably burdensome. She was almost suicidal because of having no hair on her entire body and she absolutely didn’t want to transmit this. This is a serious disorder, of which the hospital’s PGD-committee said: well, hair loss, what’s the fuss? And that in my view is the main problem, that the patient has a completely different perception than the professional. (FG-1).

When the moderators used this same case to invite discussion in the international focus group, this led to similar comments, also reflecting the notion that going ahead with such requests was sometimes needed in spite of the fact that professionals might not regard the condition serious enough to warrant PGD.

I think if the person doesn’t mind having this disease, she or he will not come to you and if he comes to me for PGD, I cannot say that I’ll not do that. (FG-2).

I don’t think that for this kind of condition, any clinical geneticist would advise to do PGD in [country] because it is really not a severe situation. (FG-2).

Whereas some participants said it was not up to them (morally) to decide about this, or that (legally) they had no authority to refuse PGD-requests for conditions not forbidden by the law in their country, others suggested that, especially in settings where PGD is reimbursed, professionals may have to act as gate keepers:

I think it is our responsibility as professionals to perform the diagnostics that we do as economically as possible. We are not to waste money. And now, when I think of this case from that view, I think that, simplified, it’s a cosmetic disease. (…) If it is only a cosmetic defect, then you have to consider if it is payable or not. (FG-2).

#### Lower penetrance and/or variable expression

With regard to conditions with a lower penetrance and/or variable expression, or more generally an uncertain prognosis, there was the same emphasis on the applicants’ perception of seriousness. But again there were different views on how this should affect decision-making. Some participants said that for them it was a matter of principle that professionals should leave it to well-informed applicants to decide whether a condition was PGD-worthy or not:

Well, I think that it is subjective on the family situation. If they have experiences with severe patients within the family, they are probably more likely to have a reason to want to exclude it. Basically that’s all we have to take into account as well. (FG-2).

I really find it bizarre that you would dare to think up limits for saying this is acceptable and this not. Look, what you should do is provide good information, both about the treatment and about possible certainties and uncertainties concerning the health of the future child, and then it is up to the individual… the couple, to do it or not. As a doctor to say ‘no’ to such requests …. for me that is only conceivable if you do more harm than good, and that should not be a personal perception of what you [emphasis] might do in that situation, but whether you think it would be harmful for the woman to undergo IVF. Otherwise I find this quite artificial… perhaps for that woman a risk of three percent is very grave. (FG-1).

On the other hand, there were reactions expressing unease with leaving this entirely to the applicants, as also very small risk factors might be perceived as serious:

But then, do you find that …, in principle you say that any disorder, if the couple finds it serious enough, is a reason for PGD…?. (FG-1).

A 16p13.1 duplication was found in a child, the father was a carrier and they want to do PGD to avoid transmission of the duplication. (…) I said ‘no’. Because it is a benign variant. But of course it is a risk factor for… a minor risk factor. So I don’t think that’s an indication. (FG-2).

I had a similar case […] The couple said even it is just one percent of risk and there is a possibility, why shouldn’t we avoid it? And then I was a bit…. well I disagreed with them, because in my opinion there was no risk. But then the couple was allowed to discuss with the other colleague of the board, and he accepted the case. But still…. because we manage PGD in a way that we avoid interfering with the couple’s decision. (FG-2).

A reason for not immediately going ahead with any request was that couples referred for PGD may not have really considered all implications. In line with that, participants stated that the role of professionals was to give them a clearer picture of what it meant to have PGD, also compared with other options. Even so, reasons for eventually leaving the decision with well-informed applicants were the fact that professionals often cannot give a precise prognosis and also that a couple’s personal experience with a disorder makes it understandable for professionals that these people would request PGD in order to avoid what might ‘objectively’ seem a lower risk:

Both with regard to variable expression as with reduced penetrance, what I find difficult is when you don’t know what the percentage is, as we now often have with array… deletions or duplications …. someone with a seriously affected brother or sister, who has the same [deletion or duplication], sitting completely normal in front of you. No literature data whatsoever…, yes and you can very well imagine that they want to avoid [a child with] the same [condition] as their sister, but you don’t know if the percentage is 1 or 50. I think, if that is your [the couple’s] experience, and you know that is indeed what may happen to you, then I find this a reasonable request for PGD. Then, for me, the precise percentage is not really important. (FG-1).

In the Dutch focus group, the same points were made with regard to disorders for which some form of treatment is available, which according to the Dutch PGD-regulation is a further factor relevant to determining ‘seriousness’. Participants stressed that treatment regimens may still come with a considerable impact on quality of life and that in times of austerity measures people may rightly be concerned about whether costly medication will continue to be reimbursed. Here again, it also made a difference when professionals considered the story behind the request as recognizable:

If a patient with long QT syndrome (LQTS) says ‘I have lost my earlier child to cot death and that’s how I found out about LQTS’, yes then we will be more inclined to [allow PGD] than when in that family they all have long lives and obediently take their beta blockers. Yet that is strange in a way. For another applicant could still say ‘I find this very threatening even though everybody is healthy’. (FG-1).

#### Transgenerational health risks

A final topic in the discussion about acceptable reasons was whether professionals would be willing to go ahead with a PGD-request for a transgenerational health risk. This was introduced with the following case that was only discussed in the international focus group. A couple where the male partner has haemophilia requests PGD in order to selectively transfer a male embryo. The reason for their request is to avoid having a daughter who (as an obligate carrier) would be at a high risk of transmitting the disorder to a further generation and thus might face the same challenges with reproductive decision making as her parents (de Wert 2005). Several participants said such requests should not be allowed as in their view that would amount to a form of eugenics:

I don’t think that it is acceptable to perform PGD just to prevent a carrier embryo, so… this is… What is the reason why we apply PGD? It is to prevent … a genetic disorder at least, but here we are not talking about preventing that or a pathogenic disorder. This is even worse than the previous case that was about the hair loss disease, at least there we have a disease that we can discuss about. (….) It will be different if the carrier will show mild signs of the disease…. This may be acceptable in my view. But if it is just about a carrier without any sign of disease, personally I refuse PGD. (…) In [country], we interpret it as eugenics. (FG-2).

If we do it once, we’ll do it every time, and then we can erase haemophilia from the earth and I think that we are not allowed to push evolution in this way. We are not gods. (FG-2).

Not all participants were as afraid of the spectre of eugenics in this context. Some participants accepted that what they were doing could be considered eugenics but protested against an all too negative view of ‘avoiding future disease’.

I think we have been haunted by the eugenics concept. (..) Of course we are improving the race, in the sense that we are avoiding someone that we consider not that healthy, or [in this case] a future disease. (...) [In this sense] we already started eugenics as soon as trisomy 21 screening was accepted. (FG-2).

One participant explained how taking the applicants’ perspective had led her to reconsider her earlier objections against allowing requests for transgenerational PGD:

In fact I remember a meeting of the European Society of Human Genetics where a similar theme was brought up, and I personally expressed that I did not think that we should offer PGD to prevent unaffected carriers being born. OK? And some people jumped on me, and said, you are obviously not from a family that ever had a problem with such condition. And it is not… you know this again brings us back to the argument, that if the family feels that their quality of life has been compromised by having this disease hanging over their heads….. then again, perhaps, with PGD which is certainly a less severe intervention than prenatal diagnosis, because what you are doing is not implanting embryos, many of which will not be implanted anyway in an IVF cycle. (FG-2).

The comparison between PGD with prenatal diagnosis (PD) as a more burdensome or ethically more sensitive procedure was repeatedly made in both focus groups. In the international focus group, PD often served as a legal reference standard: if PD (and pregnancy termination in case of a positive result) is allowed for a certain condition, then PGD should be allowed as well. In the Dutch focus group, some participants observed that whereas couples more easily tend to decide to have PGD for a certain condition, because it does not involve termination of pregnancy, current regulations in The Netherlands are stricter for PGD than for PD (where decision-making is left to individual practitioners).

### Welfare of the child

In the second part of the focus group meetings, discussions moved to welfare-of-the-child concerns in PGD-practice and the question of how to manage them.

#### PGD for affected applicants

A first issue was how professionals thought about PGD in cases where the target disorder might have a negative impact upon the parenting competence of affected applicants. An example of this is when PGD is requested for Huntington’s disease and the mutation carrier already shows signs of this devastating neurodegenerative disorder. Could this be a reason for professionals not to go ahead with a PGD-request?

In the international focus group, participants strongly agreed that this was not something for them to decide, pointing out that natural reproduction may lead to children being born in even worse circumstances.

You are allowed to have children if you are an alcoholic or drug user and yet we can’t decide if we will allow this couple to have children because they have a genetic disease…? (FG-2).

Reactions in the Dutch focus group were less dismissive of this idea. It was pointed out that ART-professionals are actively involved in creating the child, while in natural reproduction this is not so. Those explicitly speaking of a professional responsibility in this regard did so with specific reference to a guidance document of the profession (NVOG). For instance:

I find it problematic if politics tells me that I am not allowed to collaborate [with specific requests]. But reversely, I also find it a problem if I am not allowed to have a view on the matter. Of course this is a perennial issue in assisted reproduction and NVOG has issued a position statement about it. This includes that at least one should, say, ascertain that no severe harm is done to the child. And otherwise in [our] hospital there are pediatricians who will subtly remind us of this (FG-1).

When a participant who also referred to this guidance was asked what she thought of this herself, she said she agreed with the notion that professionals in assisted reproduction have a responsibility to take account of the welfare of the child:

Well, yes I do think that one… that there should be some kind of assessment, eh of the treatment that you will perform and what the consequences will be, in such a family and for the child. Yes. I can agree with all that. (FG-1).

As participants made clear, this assessment was primarily a matter of discussing the welfare of the child perspective with prospective parents, in order to find out whether they had thought about this, or invite them to do so:

I think that with all kinds of diseases [that will affect applicants] we are very much focused on how do people handle this themselves. Are they already thinking about it, about …. ‘well, yes I have this disease, so how am I going to do this with a child…’. And if we have the feeling that these people have done some good thinking about this, … have organized the necessary support, then in most cases [a treatment will follow]. (FG-1).

A good example is …. we have a patient, where the woman has osteogenesis imperfecta, but the man is also wheelchair dependent, so they are both wheelchair dependent and those people enter our room both with their wheelchair… And our first reaction is: good heavens… Until we hear that they have a completely adapted house…. that they come to us very well prepared, saying we are now ready for a child… We will soon treat them, yes. (FG-1).

One participant in the Dutch group said that in his centre they had sometimes rejected PGD specifically for Huntington’s in view of expected burdens for the child to be. But this seemed an exception. It was said that if it turned out in problematic cases that applicants had not really thought about all implications, they themselves tended to retreat their request after proper information. The view that professionals may be expected to check or raise applicants’ awareness with regard of the welfare of the child was also put forward in the international focus group, but only tentatively:

Maybe we should try to be sure that they are aware of the problem, that they know that this [the child being confronted with a parent suffering from Huntington] could happen. (FG-2).

#### Transfer of affected embryos

A second welfare-of-the-child issue in PGD may arise with respect to selecting embryos for transfer. In both groups a case was presented about an infertile couple undergoing ICSI/PGD treatment in order to have a child without the disorder they are at risk for. In this case it turns out that the last hormone-stimulation cycle they are willing or able to attempt yields only one morphologically good embryo that unfortunately happens to be an affected carrier of the disorder for which PGD was requested. The couple then asks this embryo to be transferred anyway, as—they say—it is their last chance of having a child that is genetically related to both partners.

In both focus groups, the reasoning behind such requests was discussed. Is this a matter of couples changing their minds? And if so, is that problematic? There were different opinions about this. A participant in the Dutch focus group said it should be made clear from the outset that if this situation arose, the embryo would not be transferred, as doing so was at odds with the aim of PGD. Applicants willing to eventually accept an affected ‘last chance’ embryo should in her view go for IVF (or if fertile have a child through natural conception), but not burden professionals and society with efforts and cost *“and then if the result is not to your liking, say thank you very much but we will take [the embryo]”*.

In the international focus group, one participant was concerned that the child may not be fully accepted given that its parents initially used PGD precisely to avoid (a child with) the disorder that it will have:

I don’t know, I have to think this over very carefully… I mean these people don’t want the disease and then they change their mind just because their eagerness to have a child is more important than selecting to avoid a disease….. (FG-2).

However, other participants in both focus groups thought that it was not so much a problematic change of mind on the part of the applicants, but an understandable choice for a second priority:

They wanted to have a healthy child but at the end they realized that this is not possible and then option two came up. This is the normal way you take decisions. It’s what you also do when you are going for shopping or something. (…) So this is a step-wise process and for these applicants the conditions are now different. (FG-2).

Clear differences between both focus groups emerged in terms of how professionals should react to such requests. In the international focus group, where homozygosity for disorders such as cystic fibrosis (CF) and spinal muscular atrophy (SMA) was used as an example, one participant said that in view of such a serious disease, she would definitely *“not recommend”* transferring the embryo. But if the couple were to insist, she would do as requested: *“Yes, I can tell my opinion, but the decision will be theirs”*, thereby going along with a stepwise approach that characterised patients’ decision-making. This view was widely shared in this focus group. With reference to debates in the literature about whether people with achondroplasia should be helped to have children with the disease, it was said that *“the question is not if we like it, the question should be if they like it”*.

In the Dutch focus group, some participants thought that professionals should never give in to requests to knowingly transfer an affected embryo:

We really have had this in our hospital and we have decided not to collaborate with such requests. Because we think it is essentially different to transfer an embryo that you know is affected, as compared with not checking and not knowing [as in IVF]. (FG-1).

Others made clear that, for them, it depended on the seriousness of the condition. For instance, with regard to a neuropathy leading to a 10% chance of becoming wheelchair-dependent, one participant said:

Suppose that, depending on the story, we have accepted this as an indication for PGD, and this one affected embryo is the last one left, I personally would have no difficulty with it being transferred. And then of course you say… the next question is where do you draw the line? (…) I think that for me the line would lie with, yes, children who will die at a very young age after a metabolic disorder, or SMA. (FG-1).

When challenged by another participant asking whether she could live with the thought that a wheelchair dependent child might hold her responsible for its fate, she said that *“yes I could live with that”* while also admitting that this was indeed what made *“drawing that line”* so difficult.

A senior participant in the Dutch focus group pointed out that the transfer of affected embryos was a newly emerging issue, resulting from the widening scope of PGD-indications. He said that in the past, when PGD was only done for very serious disorders, such requests simply did not arise. *“But now that we also do it for less serious conditions, you have manoeuvred yourself in a difficult position.”*

In both focus groups, it was stressed that decisions to transfer an affected embryo should ideally not be made under time pressure. It was suggested (in the Dutch focus group) that as part of general treatment counselling, preferences and options in a possible scenario of a last chance affected embryo should be discussed well beforehand with the applicants. In the international focus group it was suggested that embryo freezing was a way to buy time for the couple to reflect on their preferences.

## Discussion

In The Netherlands, the statutory regulation for PGD specifies that PGD should only be done if there is “a high individual risk of a serious genetic disorder”^12^. For the interpretation of this requirement, the regulation provides a decisional framework referring to relevant co-determinants of seriousness (transmission risk, penetrance, impact on a person’s quality of life, age of onset, availability of acceptable options for treatment or prevention). A multidisciplinary national indications committee is given the task to assess the acceptability of new applications (for disorders for which PGD was not previously done in the country) in the light of these considerations. This resembles the situation in the UK, where providers may only test for conditions approved by the HFEA on the basis of similar criteria. In a guidance document, the HFEA clarifies that with regard to conditions that have an incomplete penetrance or variable expression, the Authority will base its approval decision on a ‘worst case scenario’, while requiring professionals to determine whether PGD would be justified in particular cases, taking account of individual factors such as the subjective views, experiences and social circumstances of the applicants^13^. Similarly, the Dutch PGD regulation says that psychosocial considerations can be used for fine-tuning in cases where PGD would be indicated on the basis of ‘medical criteria’.

As Rosamund Scott et al. have argued, this regulative approach “tries both to recognise, to some extent at least, the personal nature of the issues at stake in PGD, but also to observe limits to the acceptability of the views of prospective parents and so, ultimately, to reproductive autonomy” (Scott et al. 2007).The solution of this tension is laid in the hands of professionals in PGD practice, who are given the task to assess concrete PGD-requests by doing justice to both these perspectives. In their study of how PGD professionals in a UK centre carried out this role, Scott et al. found that “although staff were sensitive to prospective parents’ views and experiences, this did not mean that they necessarily thought that ‘anything goes’. While they were often prepared to defer to prospective patients in relation to ‘grey areas’, this was largely because they thought it would be inappropriate to judge the seriousness of a condition of which they had no experience” (Scott et al. 2007). In another paper resulting from the same research, more specifically focusing on the expansion of PGD indications under discussion at that time, it was observed that staff feared it would become increasingly difficult to manage this tension, given that respecting personal choice tended to be regarded as overriding other considerations (Williams et al. 2007).

The findings from our study on what professionals regard as acceptable reasons for PGD clearly connect with this earlier research. Although (except for the statement that public money should not be wasted) there was no explicit discussion in both focus groups of possible reasons why PGD should only be offered for serious conditions, this was also not contested or denied. In both focus groups we found strong support for the notion that, if people come for PGD to avoid a certain condition, they will have experiential reason to consider that condition, or the risk of transmitting it, as serious. By saying that (if such requests were sufficiently well-informed) this would settle the issue, some participants in both groups seemed to deny that professionals had a role with regard to determining seriousness. To the extent that others did see themselves in such a role, the emphasis was on advising and counselling rather than actual gate-keeping.

There was, however, a clear difference in this respect between the two groups. Whereas participants in the international group tended to stress the applicants’ legal right to eventually have the treatment they want (whatever the views of the professional), participants in the Dutch discussion sketched a picture of shared decision-making, where professionals would go ahead with treatment in cases where they are able to understand the reasonableness of the request in the light of the couple’s reproductive history or family experience. Clearly the policy context is decisive here. The fact that in The Netherlands, as in the UK, professionals do have a checking role with regard to requests for PGD makes it important for them to see if they can identify with the story behind such requests. In (commercial) settings where a legalized understanding of reproductive autonomy is regarded as trumping other considerations, professionals may end up as mere executives of applicants’ wishes. Some frustration about this could indeed be noted in the international focus group.

Still, it can be asked if the current Dutch (and UK) regulations are not instances of PGD-exceptionalism, given that similar regulations do not govern decision-making on prenatal diagnosis (or on other medical procedures) (De Wert and Dondorp 2013). The Dutch regulations came into force as a political answer to societal concerns about broadening indications for PGD. The ruling that the applicants’ individualized perspective “can never provide a justification for PGD in cases where [the request] is to be rejected on the basis of medical criteria” serves this purpose.^14^ This means that the scope for ‘taking the story behind the request’ as a lead for shared decision-making is in fact limited. More limited indeed than what most participants in the Dutch focus group seemed to regard as naturally implied in the professional-patient relationship.

Given the emphasis in the international focus group on reproductive autonomy as overriding professional reservations, the categorical rejection of PGD for transgenerational health risks by many participants in the same group is a remarkable finding. This inconsistency seemed driven by a concern that allowing requests beyond a narrowly understood ‘medical model’ might give society cause for criticising PGD practice as driven by eugenics. The specific case that led to these reactions (PGD requested by a couple where the male partner has haemophilia in order to avoid a carrier daughter) was discussed in a recent ESHRE Task Force document calling for a broader understanding of what may be medically motivated reasons for PGD (De Wert et al. 2014b). Although a full ICSI/PGD procedure only to avoid transgenerational health risks seems disproportional, things may be different if the couple has an indication for IVF because of a fertility problem. According to ESHRE, there is no reason to regard this as a problematic form of eugenics or sex selection (De Wert et al. 2014b).

The discussion of welfare-of-the-child considerations revealed a significant difference between the two focus groups. In the international focus group there was strikingly little support for the principle [endorsed in e.g., ESHRE guidance (Pennings et al. 2007)] that concerns about the welfare of the child are relevant to decision-making about medically assisted reproduction (MAR) and may in exceptional cases lead to a reason for refusing treatment. The question whether they thought they as professionals had a responsibility in this regard was literally laughed away with the remark that fertile people who are drug addicts or worse are not barred from natural reproduction either. This response ignores that in MAR, professional action causally and intentionally contributes to the fulfilment of the couple’s reproductive plans (Pennings et al. 2007). As the moral relevance of this difference with natural reproduction seems obvious, the question arises what explains its denial by these professionals. We can only conjecture that it serves a useful role in avoiding further complications of decision making, or that it allows professionals and centres to uphold an undisturbed service ethos in settings where IVF and PGD are commercially offered. The contrasting reactions in the Dutch focus group on this issue should then perhaps be seen against the different and quite exceptional background of a country where MAR is only available in non-commercial academic centres. As was also found by Trudie Gerrits et al. in their ethnographic study of decision-making in one Dutch IVF centre (Gerrits et al. 2013), these professionals were well aware of the relevant (inter)national guidance and also seemed to have internalised this as part of their professional ethos.

Barring exceptional cases, this did not lead to refusing PGD to affected applicants. This is in line with the specification in the relevant (ESHRE, HFEA, NVOG) guidance documents stating that refusing MAR should only be considered when there is a high risk of a seriously diminished quality of life for the child-to-be. However, awareness of professional responsibility did lead to a willingness to discuss the welfare of the child with applicants in cases where this might be an issue. This may be seen as the reverse of the above finding that several professionals found it important to be able to recognize the ‘story behind the PGD-request’. Here, shared decision-making served the opposite aim of reassuring professionals that applicants had themselves taken possible reservations into account. This is what Gerrits et al. have referred to as “mirroring concerns”. They found that this made it “easier for clinic staff to respect [applicants’] autonomy and to leave the decision making to the prospective parents” (Gerrits et al. 2013).

With regard to the logic behind requests for transferring an affected embryo in ‘last chance of a child’ situations, the view of most professionals in both focus groups was in line with literature emphasizing that people reproducing through PGD do not just want to avoid the transmission of a disorder; they also want a child (Franklin and Roberts 2006; Ehrich and Williams 2010). Whereas the traditional PGD-rule that embryos with the targeted mutation or abnormality will not be transferred (Thornhill et al. 2005) can be understood as reflecting the uncontested seriousness of traditional PGD-indications, the moral landscape has changed now that PGD is more often applied for less serious conditions. Requests for transferring an affected embryo may come from infertile couples clinging to their last chance of a child, but they may also arise in the increasingly more common context of so-called ‘combination PGD’, where embryos are tested for more than one condition (De Wert et al. 2014b). Ideally this would lead to selecting good quality embryos unaffected by all target conditions, but if in the end it turns out that no such embryos are found, and a new IVF-cycle is no option, couples may request the transfer of an embryo affected by what they consider the secondary target condition. Clearly, these developments require professionals and centres to proactively define adapted transfer policies and timely discuss relevant options with applicants (Harton et al. 2011). But the challenge arising here is not only organizational. As remarked in the Dutch focus group, handling requests for transferring an affected embryo requires professionals to decide “where to draw the line” with regard to knowingly bringing a child into the world with a certain risk of a specific disorder. Given the dismissal of the very idea of a professional responsibility to consider the welfare of the child in the international focus group, it was no surprise to find that this ethical challenge was not emphasized in that group.

## Conclusion

A shared starting point for how professionals respond to PGD requests was the notion that people who come for PGD will have their own good reasons to consider the condition they wish to avoid as serious. We also found that most professionals thought that (apart from making sure that such requests are well-informed) they also had something to say about the issue. This ranged from ‘not recommending’ to sometimes also refusing treatment. As a way of bringing both perspectives together, a form of shared decision-making was proposed. It was suggested that professionals could more easily go ahead with a specific request if they themselves were able to identify with the story behind it, and to recognize the reasonableness of the request. However, the scope for this approach is limited, both in the setting of Dutch PGD practice with its PGD exceptionalism and predetermined policy rules, and in the setting of commercially offered PGD where applicants are clients rather than patients. As the findings in the second part of our study show, guidance of e.g., ESHRE (and other professional bodies and authorities) urging PGD-professionals to consider the welfare of the child in their decision-making finds little recognition in the latter setting. Whereas the notion of a professional co-responsibility in this regard was more strongly supported in the Dutch focus group, this was understood as primarily requiring professionals to make sure that relevant concerns are given due attention by the applicants, thus as much as possible avoiding the need of putting themselves in the role of moral gate-keepers. However, moral tensions may still arise in concrete cases, leaving professionals without much specific guidance as to what their responsibility would require. Considering that as a result of the widening scope of accepted indications for PGD, professionals will more often be confronted with requests to transfer an affected embryo, this was regarded as a challenge that will become only more important in the near future.

## Limitations

The findings of this qualitative study, based on two focus group meetings in different settings, cannot be generalized to all possible settings in which professionals are confronted with PGD decisions. Also, it is important to note that this study does not provide data on actual responses of physicians to PGD-requests. However, the study does offer an in-depth discussion of the topics and criteria that were considered relevant by the participating professionals. One of the strengths of the study is the fact that the professionals were recruited based on their participation in meetings related to the topic of PGD, which lead to the participation of highly experienced professionals. Another strength of the study is the use of focus groups where participants were invited to reflect upon their own and their colleagues’ reasoning and thereby acted as co-researchers. This made it possible for them to influence the research agenda (for instance by presenting their own cases) and to invite the group to consider topics or questions that were not prepared by the researchers.

## Footnotes

1 Denmark: Bekendtgørelse af lov om assisteret reproduktion i forbindelse med behandling, diagnostik og forskning m.v. (2015) § 7. https://www.retsinformation.dk/forms/r0710.aspx?id=167647 (last visited 05-09-2017).

2 France: Code de la Santé Publique (2011) Ch I: Diagnostics antenataux : diagnostic prénatal et diagnostic préimplantatoire. Article L2131-4. https://www.legifrance.gouv.fr/affichCodeArticle.do?cidTexte=LEGITEXT000006072665&idArticle=LEGIARTI000006687397&dateTexte=&categorieLien=cid (last visited 05-09-2017).

3 Germany: Embryonenschutzgesetz § 3a Präimplantationsdiagnostik; Verordnungsermächtigung (2011). http://www.gesetze-im-internet.de/eschg/__3a.html (last visited 05-09-2017).

4 Netherlands: Regeling pre-implantatie genetische diagnostiek (PGD) (2009). http://wetten.overheid.nl/BWBR0025355/2013-04-06 (last visited 05-09-2017).

5 Norway: Bioteknologiloven (2003) § 2A-4 Behandling av søknader om preimplantasjonsdiagnostikk. https://lovdata.no/dokument/NL/lov/2003-12-05-100 (last visited 05-09-2017).

6 Sweden: Lag om genetisk integritet m.m. (2006) 4 Kap Fosterdiagnostik, genetisk fosterdiagnostik och preimplantatorisk genetisk diagnostik § 2. http://www.riksdagen.se/sv/dokument-lagar/dokument/svensk-forfattningssamling/lag-2006351-om-genetisk-integritet-mm_sfs-2006-351 (last visited 05-09-2017).

7 UK: Human Fertilisation and Embryology Act (2008) 22 /schedule 2 /paragraph 1ZA. http://www.legislation.gov.uk/ukpga/2008/22/schedule/2 (last visited 05-09-2017).

8 Spain: Ley sobre técnicas de reproducción humana asistida (2006). Art 12 Diagnóstico preimplantacional. http://noticias.juridicas.com/base_datos/Admin/l14-2006.html#cpa12 (last visited 05-09-2017).

9 Belgium: Wet betreffende de medisch begeleide voortplanting en de bestemming van de overtallige embryo’s en de gameten. Titel VI Hoofdstuk II Art 67 (2007). http://www.ejustice.just.fgov.be/cgi_loi/change_lg.pl?language=nl&la=N&cn=2007070632&table_name=wet (last visited 05-09-2017).

10 Human Fertilisation & Embryology Authority (HFEA). Code of practice #8 (2017). Welfare of the Child.https://www.hfea.gov.uk/code-of-practice/8 (last visited 05-09-2017).

11 Nederlandse Vereniging voor Obstetrie en Gynaecologie (NVOG). Modelprotocol mogelijke morele contra-indicaties bij vruchtbaarheidsbehandelingen (2010). http://nvog-documenten.nl/richtlijn/doc/index.php?type=save&richtlijn_id=864 (last visited 05-09-2017).

12 Cf note 4.

13 Human Fertilisation & Embryology Authority (HFEA). PGD application process. http://hfeaarchive.uksouth.cloudapp.azure.com/www.hfea.gov.uk/8865.html2014 (last visited 05-09-2017).

14 Cf note 4.
